# A Novel Equivalent Time Sampling-Based Method for Pulse Transit Time Estimation with Applications into the Cardiovascular Disease Diagnosis

**DOI:** 10.3390/s23115005

**Published:** 2023-05-23

**Authors:** Giorgia Fiori, Fabio Fuiano, Silvia Conforto, Salvatore Andrea Sciuto, Andrea Scorza

**Affiliations:** Department of Industrial, Electronic and Mechanical Engineering, University of Roma Tre, 00146 Rome, Italy

**Keywords:** color Doppler, equivalent time sampling, flow phantom, arterial simulator, flow average velocity, pulse wave velocity, image analysis, pulse transit time

## Abstract

The increasing incidence of cardiovascular diseases (CVDs) is reflected in additional costs for healthcare systems all over the world. To date, pulse transit time (PTT) is considered a key index of cardiovascular health status and for diagnosis of CVDs. In this context, the present study focuses on a novel image analysis-based method for PTT estimation through the application of equivalent time sampling. The method, which post-processes color Doppler videos, was tested on two different setups: a Doppler flow phantom set in pulsatile mode and an in-house arterial simulator. In the former, the Doppler shift was due to the echogenic properties of the blood mimicking fluid only, since the phantom vessels are non-compliant. In the latter, the Doppler signal relied on the wall movement of compliant vessels in which a fluid with low echogenic properties was pumped. Therefore, the two setups allowed the measurement of the flow average velocity (FAV) and the pulse wave velocity (PWV), respectively. Data were collected through an ultrasound diagnostic system equipped with a phased array probe. Experimental outcomes confirm that the proposed method can represent an alternative tool for the local measurement of both FAV in non-compliant vessels and PWV in compliant vessels filled with low echogenic fluids.

## 1. Introduction

In the last few years, the study of the main mechanisms involved in the cardiovascular system has been especially prolific [1,2,3,4,5,6,7]. This attention can be explained by the recent publication of the Global Health Observatory data, by the World Health Organization (WHO), on death estimates based on cause, age, sex, country and region [8]. The statistics on the causes of death help the sanitary authorities to determine the objective of their actions in public health [9,10]. Among the top ten world death causes in 2019, heart diseases and ischemic ictuses can be found [8]. Therefore, the prediction of cardiovascular diseases (CVDs) assumes a key role. Such issues fully justify the increasing attention in the development of models able to study the most predictive parameters of such diseases and in the investigation of novel risk indicators. Among them, several studies highlighted interest in the estimation of the pulse transit time (PTT), defined in the literature as the time delay between two different recording sites of a homogeneous medium in which a pressure wave propagates [11,12]. It is nowadays an accepted marker of cardiovascular health status as its variations have been found to be related to changes in cardiovascular hemodynamics, e.g., arterial stiffness, blood pressure and heart rate [13,14,15]. In this regard, several studies [16,17,18,19,20,21,22] can be found assessing PTT both in vivo and in vitro although, to the best of the author’s knowledge, none of these use an automated image analysis approach [16,17,18,19,20,21,22]. Alongside pulse transit time, pulse wave velocity (PWV), i.e., the velocity of the cardiac pressure pulse in arteries, is commonly considered a predictive diagnostic parameter for CVDs [23,24]. In particular, PWV is a physical quantity closely related to the mechanical status of arteries and their functioning [2,24]. Nowadays, it is assessed as the ratio of the distance between two measurement sites and PTT, allowing an indirect estimation of the blood vessel stiffness. In this regard, it is noteworthy to highlight that PWV measurement uncertainty is lower for invasive and local measurements, while it is higher for non-invasive ones, mainly because of the difficulty in accurately evaluating the distance [12,25]. In addition, it must be taken into account that the mechanical and geometric characteristics of arterial vessels change depending on the proximity to the heart (e.g., aorta or peripheral vessels), greatly affecting PWV [24,26]. This leads to the necessity of developing novel, non-invasive, local and accurate measurement methods. Among all the non-invasive devices available on the market [12,23,27], ultrasound (US) systems are widely used in clinical practice for screening, diagnosis and therapy. In this context, the American Society of Echocardiography included cardiovascular US technology development for prognostic assessment in patients with CVDs as one of the main goals in the roadmap for 2020 [28]. As regards ultrasonographic techniques, pulsed wave Doppler has been proposed in recent years for PWV measurement [24,27,29]. The measurement is carried out sequentially by recording the Doppler waveform in two sites of the artery and estimating the time delay between the pulse waves, while the path length is assessed on the body surface as the distance between the recording sites. The main drawbacks are the regional distance estimation (i.e., over a long arterial segment) and the non-simultaneous recording of the two Doppler signals whose synchronization requires ECG acquisition [27,29]. Moreover, common US equipment shows bandwidth limits due to the image acquisition frequency (or frame rate) which makes it impossible to detect waveforms that rapidly vary over time, as in the case of PWV (i.e., from about 5 m·s^−1^ until over 15 m·s^−1^) in clinical practice [30].

In this context, the present study aims at proposing a novel image analysis method, based on equivalent time sampling (ETS), named equivalent time sampling-based image analysis method (ETiSIAM), which has revealed itself able to alternatively estimate (a) the flow average velocity (FAV) in a non-compliant vessel and (b) PWV in a compliant vessel through the pulse transit time assessment. This was possible because the proposed method is independent from the physical phenomenon determining the Doppler signal, since the latter can be generated, alternatively, by (a) the flow of an echogenic material and (b) the vessel wall movement, according to the measurement setup.

In the case of a non-compliant vessel, the method was applied for FAV estimation and tested on a Doppler flow phantom, set in pulsatile mode at different phantom frequencies and flow regimes. Tests were carried out in dynamic conditions where aliasing can occur because of technological limitations of the instrumentation. The FAV estimation was carried out locally because ETiSIAM allows PTT investigation on a region of interest smaller than in other methods, adjusted by the operator on the displayed vessel.

On the other hand, in the case of a compliant vessel, the method was applied for PWV estimation and tested on an ad hoc arterial simulator. The latter was mainly constituted by an elastic hose as an arterial surrogate, inserted in a hydraulic circuit where pulses were provided by a peristaltic pump. Moreover, it included an integrated system, able to change the mechanical properties of the arterial surrogate as in [31]. Notably, in the current state of the art, US systems have been employed both in A-mode and Doppler imaging on arterial simulators to retrieve information about the arterial surrogate diameter and wall thickness, or the cross-section flow velocity [32,33]. Nevertheless, none have been used as a tool to estimate PWV in different arterial surrogate conditions, nor has a solution to the limitation on the US frame rate been proposed.

In both cases, PTT was estimated through the processing of color Doppler (CD) videos collected from an ultrasound diagnostic system equipped with a phased array probe. In this regard, this study constitutes a novelty in the current scientific field. Up to now, ETS has been applied in the biomedical field as a sampling technique in the pre-acquisition phase [34,35] and it seems that it has rarely been used as a post-processing tool. To the authors’ knowledge, just a single study proposing a first approach for the ETS reconstruction of the electrocardiographic signals in the post-processing phase has been carried out [36]. The novelty of the present study is the ETS post-processing application to CD videos acquired from two different experimental setups, to demonstrate its potentiality in the US diagnostic field for the assessment of both FAV and PWV, according to the characteristics of the test object under investigation and overcoming the bandwidth limitations of the ultrasound system.

The study presented in this paper is organized as follows: in Section 2, the two experimental setups are described together with the main processing steps of the proposed image analysis method based on the equivalent time sampling. Section 3 deals with the measurement uncertainty analysis through Monte Carlo simulations. Experimental results retrieved for both experimental setups are presented in Section 4, while in Section 5, they are discussed, highlighting future research directions. Finally, conclusions are outlined in Section 6.

## 2. Materials and Methods

### 2.1. Experimental Setup

#### 2.1.1. Flow Phantom

Doppler 403^TM^ Flow Phantom [37] is a commercial self-contained reference device, constituted by a tissue mimicking material (TMM) as phantom background embedding a continuous non-compliant flow circuit with a horizontal (at 2 cm depth) and a diagonal (at 40° from 2 to 16 cm depth) tube segment containing the blood mimicking fluid (BMF), and finally, an electric flow controller providing constant or pulsatile flows up to 12.5 mL·s^−1^. The main characteristics of the device are reported in Table 1. In this study, the device was set to provide a pulsatile flow:

1. at two pump frequencies (i.e., 1.3 and 2.0 Hz) and five flow regimes (i.e., 2.0, 4.0, 6.0, 8.0 and 10 mL·s^−1^);
2. at three further pump frequencies (i.e., 0.5, 0.8 and 1.0 Hz) by maintaining a flow regime of 8.0 mL·s^−1^.

Color Doppler data were collected through a US diagnostic system equipped with a phased array probe, whose main settings are listed in Table 2. Based on [36], a Doppler video lasting 140 s was acquired for each phantom frequency and flow regime and post-processed by ETiSIAM for PTT estimation. Then, FAV value was assessed and compared with the corresponding preset value.

#### 2.1.2. Arterial Simulator

**Figure 1:**
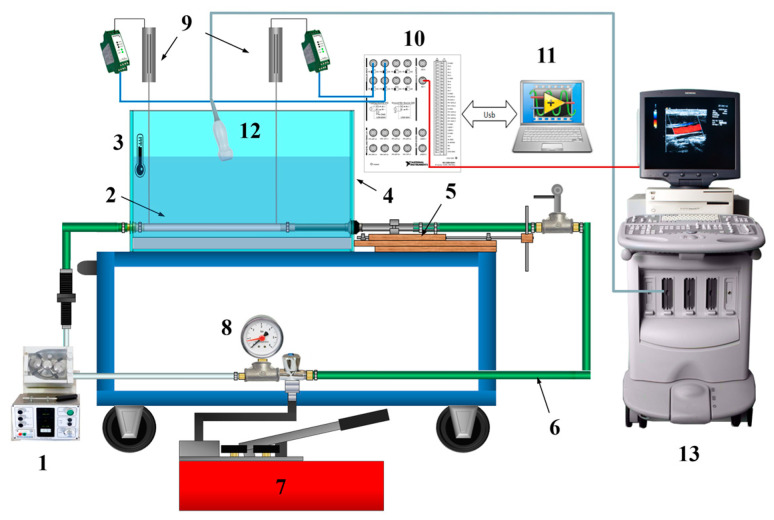
Arterial simulator experimental setup scheme: (1) peristaltic pump, (2) arterial surrogate, (3) bulb thermometer, (4) water tank, (5) tensioning state, (6) hydraulic circuit, (7) manual pump, (8) analog manometer, (9) LVDTs, (10) NI-DAQ device, (11) personal computer, (12) ultrasound probe, (13) ultrasound diagnostic system. The probe holder is not represented.

The experimental setup previously developed in [31] was adopted in this study for PWV measurements. The operating principle of such a simulator is based on the mathematical model investigated in [21,38]. The components constituting the setup, shown in Figure 1, are the following:

(1) a peristaltic pump providing pulses to the system to reproduce the pumping function of the heart. A higher pumping frequency was chosen in order to avoid the resonance frequency of the whole system;
(2) a silicone hose as an arterial surrogate;
(3) a bulb thermometer for temperature monitoring since this physical quantity could affect PWV measurements;
(4) a tank filled with distilled water used to (a) house the arterial surrogate, (b) allow the US probe imaging of the surrogate and (c) simulate a physiological environment;
(5) a tensioning system to simulate PWV variations, equipped with a graduated scale for the hose stretching;
(6) a rigid hose to complete the hydraulic circuit;
(7) a manual pump to fill the whole hydraulic circuit with a low echogenic mixture and put it under pressure;
(8) an analog manometer to monitor the inner pressure of the hydraulic circuit, and consequently, the arterial surrogate;
(9) two general-purpose LVDT sensors to record the radial displacement waveform in two recording sites on the arterial surrogate;
(10) a National Instruments-Data Acquisition (NI-DAQ) device, model USB-6341, for LVDT waveforms acquisition;
(11) a personal computer for data storage;
(12) a phased array probe to detect and display the CD signal;
(13) a US system for color Doppler imaging and data acquisition.

Further characteristics of the simulator components are listed in Table 3. In this study, six tensioning states were applied to the arterial surrogate at a peristaltic pump frequency of 3.4 Hz. The acquisition settings listed in Table 4 were maintained throughout the measurement campaign.

The abovementioned US diagnostic system was used to acquire CD data from the arterial simulator without changing the system settings (Table 2) and simultaneously for LVDT signals acquisition. The wall filter setting adjustment at the minimum level allowed the detection of the wall movement artifact of the surrogate. The acquired data were post-processed by ETiSIAM for PTT estimation in order to assess the PWV and compare it with the outcomes retrieved through the LVDT signals processing.

As shown in Figure 1, the phased array probe was placed on the water surface in a fixed position using a dedicated holder to guarantee clear visibility of the surrogate. Moreover, the probe was tilted to avoid the directional ambiguity artifact [39]. Indeed, to display the color Doppler flow due to the radial velocity of the hose, the perpendicular orientation must be avoided, due to the Doppler intrinsic limitations.

### 2.2. Equivalent Time Sampling-Based Image Analysis Method (ETiSIAM)

**Figure 2:**
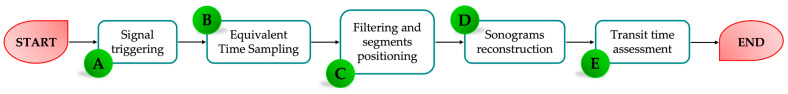
Flow chart of ETiSIAM for pulse transit time assessment.

The equivalent time sampling-based image analysis method post-processes color Doppler videos, collected from duplex systems, to assess the pulse transit time between two recording sites on a vessel. ETiSIAM was implemented in a MATLAB environment through an ad hoc procedure to reconstruct a single period of a periodically varying waveform due to pumping action. In particular, ETiSIAM allows the reconstruction of two temporal evolutions, called sonograms [40], assessed in two sites at a fixed distance *L* on the vessel. From the sonograms, PTT, i.e., the time delay in the pulse propagation, can be estimated. The main steps of ETiSIAM are described in the following and shown in Figure 2.

The choice of CD mode in ETiSIAM allows focusing the transit time investigation on a smaller region of interest because of the higher sensitivity on velocity rather than displacements, whose measurements in B-mode are in turn affected by low spatial resolution. Therefore, ETiSIAM may also be used to overcome the current limits in sensitivity, resolution and frame rate of US diagnostic systems in the detection of rapid time-varying phenomena. This limitation primarily affects obsolescent ultrasound systems, especially when used in CD mode.

#### 2.2.1. Signal Triggering

ETiSIAM was developed starting from a previous study [36], in which ETS was applied to electrophysiological signals. As already described in [36], ETS relies on a trigger event to collect samples. Therefore, a periodic or pseudo-periodic signal must be injected into the US system through its ECG module. The latter consists of several physiologic channels, or patient connections, including not only ECG but also phono (heart-sounds), respiration and pulse. By considering the two experimental setups included in this study, the chosen trigger events were provided by:

o a sinusoidal waveform, generated by a signal generator, whose frequency was set equal to the nominal flow pumping frequency;
o one of the two LVDT waveforms, processed in real-time (i.e., offset removal, amplification and low pass filtering) through an in-house LabVIEW VI.

For both setups, a trigger event occurred whenever ETiSIAM detected a waveform peak in the CD frames. It is worth noting that in a real-case scenario, the trigger will be provided by the ECG signal of the patient acquired simultaneously with the Doppler video. Therefore, the trigger event will be the R-peak of the QRS complex.

The automatic peaks detection is carried out by applying a color level threshold *th_C_* equal to 30% of the maximum value of the pixels contained in the image ECG box. Both the index identifying the frames where a peak is found and the time instant of each peak *T_p_* are consequently stored.

#### 2.2.2. Equivalent Time Sampling

The number of detected peaks *N_p_*, is the maximum number of samples, each one collected in correspondence with a trigger event that can be used for the reconstruction of the signal period *T*. Therefore, the time interval Δ*τ* between two consecutive samples can be derived as:

(1) $$\Delta \tau =\frac{T}{N_{p}}$$

Consequently, the time instant *t_samp_* of the CD frames to be sampled is identified as follows:

(2) $$t_{samp}=T_{p}+m\Delta \tau$$

where *m* is an integer number.

#### 2.2.3. Filtering and Segments Positioning

**Figure 3:**
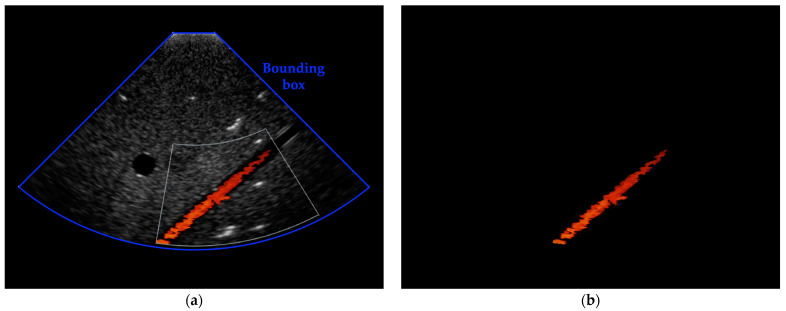
(**a**) Example of bounding box extraction in the first sampled frame before threshold-based filter application; (**b**) corresponding filtered frame containing the color Doppler information only.

The bounding box containing the diagnostic image is extracted (excluding patient and settings details) and then processed by a threshold-based filter in order to remove the B-mode information and leave the CD information only [41,42,43], as shown in Figure 3. These processing steps are applied to all the sampled frames.

At this point, ETiSIAM requires the computation of the flow central axis and its inclination for the automatic positioning of two groups of segments intersecting the flow at 90°. Such a procedure is carried out for the first sampled frame only and the retrieved axis coordinates are used for all the other sampled frames to avoid unwanted segment mispositioning and to reduce the computational cost.

**Figure 4:**
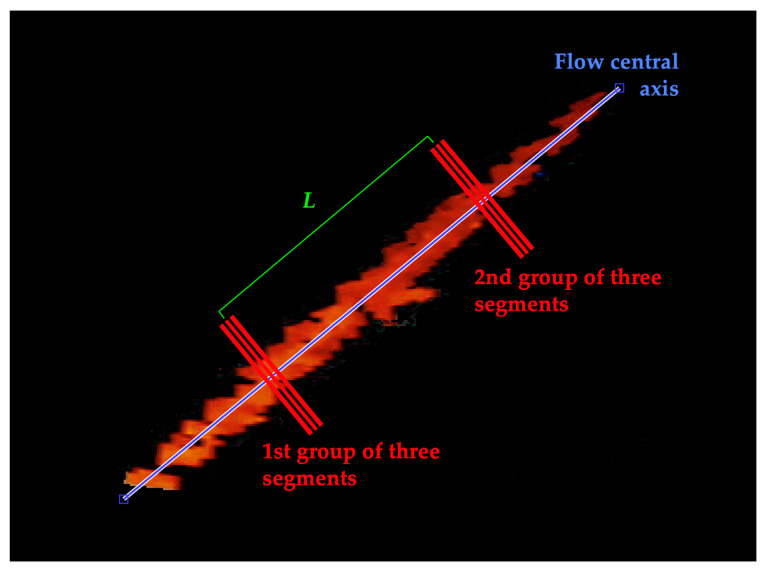
Example of segments positioning on the flow central axis in the first sampled frame. For every group of (three) segments, a sonogram is reconstructed.

Afterwards, the first group of three segments is positioned perpendicularly to the axis at 30% of its total length to guarantee a safety margin. Similarly, the second group of three segments is placed at a fixed distance *L* from the first group (Figure 4). The length of the segments is set to be higher than the nominal diameter of the vessel.

#### 2.2.4. Sonograms Reconstruction

**Figure 5:**
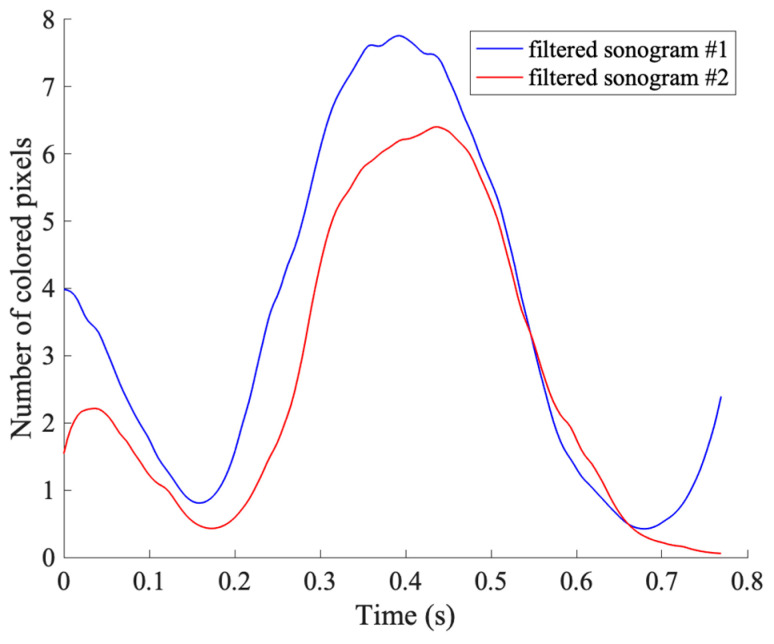
Examples of filtered sonograms retrieved through the flow phantom experimental setup: 1.3 Hz phantom pump frequency at a flow regime of 10 mL·s^−1^.

In the last step of the image processing, the number of colored pixels for each segment is computed. To avoid the counting of any pixels erroneously included and not belonging to the flow (e.g., due to filtering errors), the mean number of colored pixels for each group is retrieved. This computation is repeated for all the sampled frames and the sonograms (i.e., the mean number of colored pixels variation over a period among the three segments) are obtained by the time alignment of all the mean number of colored pixels with Δ*τ* spacing. Finally, the two obtained waveforms are filtered through a low-pass filter, as shown in Figure 5. The cut-off frequency is adjusted according to the frequency content of the trigger signal, i.e., pumping frequency.

#### 2.2.5. Transit Time Assessment

In the last step of the method, the time delay between the two sonograms *S*′ and *S*″ is computed through the correlation technique, as follows:

(3) RS′S″(PTT)=limT→∞[1T∫−T2T2S′*(t)·S″(t+PTT) dt]

where *R_S_*_′*S*″_ quantifies the correlation degree between *S*′(*t*) and *S*″(*t*) when the two distinct events are observed in instants shifted by a time delay equal to PTT.

The method specifications assumed in this study for the two experimental setups are listed in Table 5.

### 2.3. FAV and PWV Estimation

At this point, the pulse transit time for the flow phantom experimental setup (*PTT_ph_*) can be used to estimate the flow average velocity as follows:

(4) $$FAV=\frac{L_{ph}}{PTT_{ph}}\;$$

where *L_ph_* is the fixed distance between the two recording sites on the phantom vessel. In this case, data validation was carried out through the comparison with the nominal flow velocities. As already adopted in [44], compatibility is guaranteed if the following inequality is satisfied [45]:

(5) $$\left|\mu_{1}-\mu_{2}\right|\leq \sigma_{1}+\sigma_{2}$$

where *μ*_1_ is the mean FAV value estimated through the proposed image analysis-based method, while *μ*_2_ can either be: (a) a second estimated mean FAV value at a different pump frequency, or (b) the corresponding nominal mean FAV value provided by the phantom datasheet. On the other hand, *σ*_1_ and *σ*_2_ are the corresponding standard deviation values.

Similarly, the pulse transit time for the arterial simulator experimental setup (*PTT_as_*) can be used to estimate the pulse wave velocity as follows [12,22]:

(6) $$PWV=\frac{L_{as}}{PTT_{as}}\;$$

where *L_as_* is the fixed distance between the two recording sites on the arterial surrogate adjusted on the US image. In this second case, compatibility is evaluated by applying Equation (5), in which *μ*_1_ is the mean PWV value estimated through the proposed image analysis-based method, while *μ*_2_ is the corresponding value retrieved through the two LVDT signals. The latter were processed as in [31] by applying band-pass filtering and adaptive thresholding for the estimation of the transit time between the LVDTs recording sites. Then, the pulse wave velocity *PWV_LVDT_* was computed as in Equation (6) but considering the physical distance between the LVDT rods over the arterial surrogate (see Table 4).

## 3. Monte Carlo Simulation

In recent literature, Monte Carlo Simulation (MCS) has been extensively used for the uncertainty evaluation of measurements carried out through software-based methods [38,41,44,46]. Two different MCS series were carried out for the uncertainty evaluation related to the estimation of FAV and PWV on the Doppler phantom and the arterial simulator, respectively. As regards the former, an MCS (10^5^ cycles) was carried out for each phantom frequency-flow regime pair to evaluate the FAV standard deviation (SD). For the latter, a MCS (10^5^ cycles) was repeated for all the tensioning states applied to the arterial simulator to estimate the PWV standard deviation.

### 3.1. Uncertainty Analysis: Flow Phantom

The following uniform distributions (Table 6) were assigned to the variables influencing FAV, according to Equation (4): the distance *L_ph_* between the two groups of segments, affected by the pixel resolution *δ_px_*; the pulse transit time between the two corresponding sonograms, affected both by the ETS period Δ*τ* in Equation (1); and the intrinsic error due to the application of the correlation technique (correlation coefficients were always higher than 0.98). The total standard uncertainty *σ*_Δ*t*,*ph*_ was estimated as the square sum of the two contributions for each pump frequency–flow regime pair:

(7) $$\sigma_{\Delta t,ph}=\sqrt{\sigma_{ETS}^{2}+\sigma_{corr}^{2}}$$

where *σ_ETS_* was *σ_corr_* are the standard deviation values associated with the application of the equivalent time sampling and the correlation technique, respectively.

### 3.2. Uncertainty Analysis: Arterial Simulator

In order to evaluate the uncertainty contribution of the application of ETiSIAM to the arterial simulator, the following distributions (Table 7) were assigned to the physical quantities in Equation (6): a uniform distribution to the distance *L_as_* between the two groups of segments on the arterial surrogate image, which is mainly affected by the pixel resolution *δ_px_*; and a uniform distribution for the transit time, mainly affected by (a) Δ*τ* spacing, (b) the estimation of the temporal delay, and (c) the repeatability uncertainty of the six tests performed for each tensioning state. Additionally, in this case, the total standard uncertainty *σ*_Δ*t*,*as*_ was estimated as the square sum of the single contributions according to the tensioning state, as in the following:

(8) $$\sigma_{\Delta t,as}=\sqrt{\sigma_{ETS}^{2}+\sigma_{corr}^{2}+\sigma_{rep}^{2}}$$

where *σ_rep_* is the standard deviation estimated from sixfold test repetition.

Similarly, the uncertainty contribution associated with the PWV values retrieved through the processing of the LVDT signals was estimated by assigning uniform distributions to the distance between the two displacement transducers (45.5 ± 0.6 cm) and to the transit time varying according to the tensioning state.

## 4. Results

### 4.1. Flow Average Velocity Results

**Figure 6:**
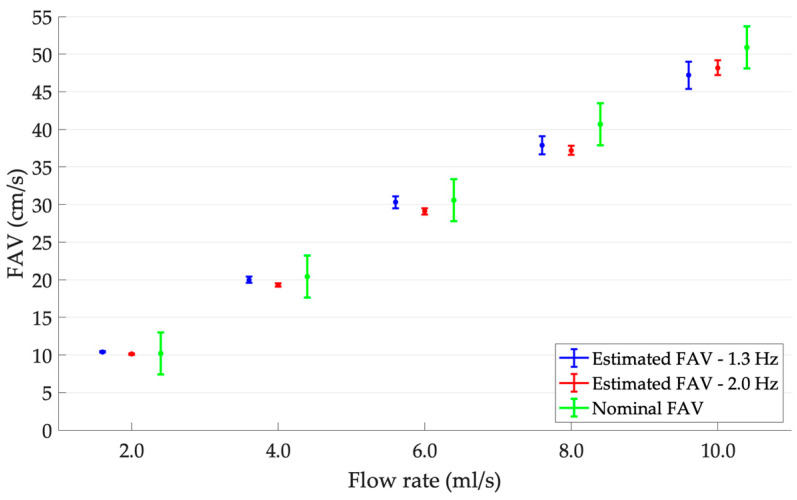
FAV outcomes with error bars for increasing flow rates at 1.3 and 2.0 Hz pumping frequencies compared with the nominal FAV values.

Flow average velocity results (mean ± SD) obtained through ETiSIAM application at 1.3 and 2.0 Hz pump frequencies and different flow regimes are reported in Table 8 and shown in Figure 6. The nominal mean FAV values are the ones provided by the flow phantom datasheet, while the corresponding standard deviations *σ_FAV_* were derived as follows:

(9) $$\sigma_{FAV}=FAV_{FS}\sqrt{{\left(\frac{\sigma_{Q}}{Q_{FS}}\right)}^{2}+{\left(2\frac{\sigma_{d}}{d}\right)}^{2}}$$

where *FAV_FS_* is the nominal FAV obtained from the flow rate at full-scale *Q_FS_*, *d* is the nominal tube diameter, while *σ_Q_* and *σ_d_* are the corresponding SDs. The latter were retrieved from the measurement uncertainties reported in the phantom datasheet (see Table 1), assuming a normal distribution with 95% confidence level for both quantities.

As shown in Figure 6, the compatibility between the nominal and estimated FAV values was almost always guaranteed. Moreover, FAV outcomes estimated through the application of ETiSIAM increased, as expected, by increasing the flow rate on the phantom under test. In order to quantify the normalized percentage dispersion of ETiSIAM in the FAV estimation, the relative percentage standard deviation *μ_SD_*_%_ was computed for each flow regime at 1.3 and 2.0 Hz (Table 8). It can be noticed that, independently of the phantom pump frequency, such an index increased for increasing flow rate regimes.

Finally, the percentage error *ε_FAV_*_%_ of the mean estimated FAV value (*FAV_est_*) was computed in order to quantify the discrepancy with the corresponding mean nominal one (*FAV_nom_*), as in [47]:

(10) $$\varepsilon_{FAV\%}=\frac{\left|FAV_{nom}-FAV_{est}\right|}{FAV_{nom}}\cdot 100$$

**Figure 7:**
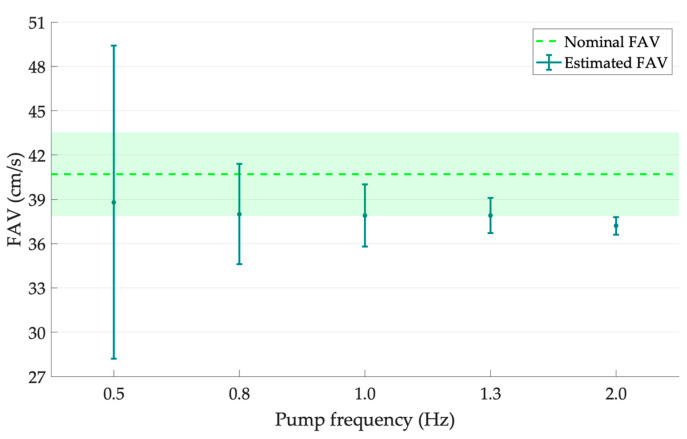
FAV outcomes with error bars for increasing pump frequencies of the Doppler phantom at 8.0 mL·s^−1^ compared with the nominal FAV values.

As regards the second set of data acquired from the Doppler phantom, FAV results (mean ± SD) obtained at three further pump frequencies (i.e., 0.5, 0.8 and 1.0 Hz), by maintaining a flow regime of 8.0 mL·s^−1^, are reported in Table 9 and shown in Figure 7. The outcomes were globally compatible according to Equation (5), and therefore, as expected, they suggested that FAV did not depend on frequency. The highest standard deviation can be found in correspondence with the lowest phantom pump frequency because of the lowest ETS frequency due to the limited number of peaks acquired in 140 s. In this regard, the SD values showed a decreasing trend with increasing pump frequencies and a relative percentage standard deviation *μ_SD_*_%_ from 27.3% to 1.6%. This was probably due to the increasing equivalent time sampling frequency. In fact, the number of trigger events (i.e., peaks) was limited by both the pump frequency and the acquisition time. Finally, the percentage error *ε_FAV_*_%_, computed according to Equation (8), was almost constant for a pump frequency ranging from 0.8 to 1.3 Hz, with the highest value at 2.0 Hz.

### 4.2. Pulse Wave Velocity Results

**Figure 8:**
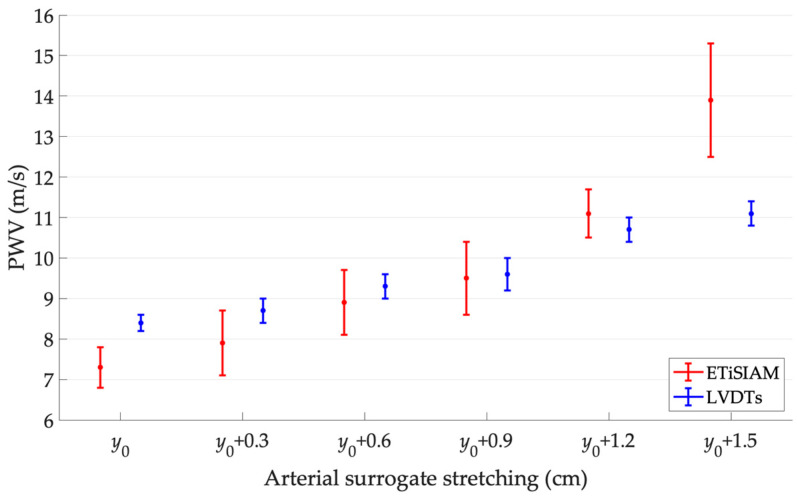
PWV outcomes with error bars for increasing tensioning states.

Pulse wave velocity outcomes (mean ± SD) retrieved for the arterial simulator experimental setup are reported in Table 10 and shown in Figure 8. As already found in [31], the arterial surrogate stretching causes a transit time decrease, which in turn can be associated with a stiffness increase, related to an increase in PWV. Such behavior seems to be confirmed by the outcomes obtained both from the image analysis-based method and the LVDTs.

It is also possible to establish a linear relationship between the different surrogate tensioning states and the PWV variation through the least squares method application. The straight lines that best approximate the two trends in Figure 8 show a positive angular coefficient with a correlation coefficient R^2^ = 0.95 and R^2^ = 0.98 for ETiSIAM and LVDTs, respectively.

The PWV standard deviations retrieved through the LVDTs processing do not show significant variations for increasing tensioning states of the arterial surrogate. On the other hand, the ones estimated through ETiSIAM seem to increase with the increased stretching of the arterial surrogate. In order to quantify the normalized percentage dispersion of ETiSIAM in the PWV estimation with respect to the displacement transducers, the relative percentage standard deviation *μ_PWV_*_,*SD*%_ was computed for each tensioning state (Table 10).

Results compatibility was tested according to Equation (5). It can be observed that PWV measurements (Table 10) showed compatibility for intermediate stretching states, while a significant discrepancy was found for extremal measurement conditions. This discrepancy could be probably due to inertial limitations introduced by the LVDT transducers, i.e., the weight of cores and rods on the arterial surrogate.

On the basis of the scientific literature, PWV changes according to the health status of the arterial tree, assuming values until 13–15 m·s^−1^ for pathological or aging conditions [48,49,50,51]. In this regard, the arterial simulator seemed to be able to simulate PWV variations mostly in the pathological range. Such variations were detected through both the LVDTs and ETiSIAM herein proposed and applied to color Doppler image analysis.

Finally, the percentage error *ε_PWV_*_%_ of the mean *PWV_ETS_* value was computed, as already done for the previous setup, in order to quantify the discrepancy with the corresponding mean *PWV_LVDT_* value, as follows:

(11) $$\varepsilon_{PWV\%}=\frac{\left|PWV_{LVDT}-PWV_{ETS}\right|}{PWV_{LVDT}}\cdot 100$$

The highest percentage error was found in correspondence with the higher surrogate stretching, while the lowest with the intermediate tensioning state.

## 5. Discussion

The present study focused on a novel image analysis method based on equivalent time sampling, named ETiSIAM, for pulse transit time estimation. It represents the first attempt to use ETS as a post-processing tool applied to ultrasound diagnostic data. The method processes color Doppler videos by applying the following main steps: signal triggering, equivalent time sampling, filtering and segments positioning, sonograms reconstruction, and finally, transit time assessment. As a first approach, it was tested on two experimental setups for two different non-invasive measurement applications. A commercial Doppler flow phantom, constituted by non-compliant vessels, was used for flow average velocity assessment, whilst an ad hoc arterial simulator, consisting of a compliant arterial surrogate filled with a low echogenic mixture, was used for local pulse wave velocity assessment. For both experimental setups, Doppler data were collected through a single US diagnostic system equipped with a phased array probe. As regards testing conditions, the flow phantom was set to provide a pulsatile flow at (a) two pump frequencies for five different flow regimes, and (b) three further pump frequencies while maintaining the same flow regime. On the other hand, six increasing stretching states were applied to the arterial surrogate to simulate different pulse wave velocity variations. Results validation was carried out through the comparison with the nominal flow velocities provided by the phantom datasheet for the first experimental setup, and with the outcomes retrieved through the LVDTs for the second experimental setup.

By focusing on the flow phantom, results retrieved through ETiSIAM were globally compatible for each pump frequency-flow regime pair (Table 8 and Table 9), therefore suggesting that the proposed image analysis method can represent an alternative tool for non-invasive flow average velocity assessment in non-compliant vessels. In this case, the wall movement of the phantom vessels can be neglected, and the Doppler shift is due to the echogenic properties of the blood mimicking fluid only. In more detail, the BMF is constituted by scatterers flowing into the tube of the phantom set in pulsatile mode. Therefore, the implemented method performs color Doppler signal tracking by post-processing the acquired frames whose CD patterns are generated by the interaction of the US beam with both the BMF particles and the tube walls. Since the phantom tube is a non-compliant vessel, the detected signal is mostly from the echogenic fluid. Conversely, the arterial surrogate in the arterial simulator is a compliant vessel moving under the action of the peristaltic pump and filled with a low echogenic mixture: in this case, the detected signal is mostly from the surrogate movement. It should be noticed that in clinical practice the wall movement is commonly assumed as a motion artifact, therefore it is removed through the wall filter setting on the US scanner to prevent the artifact from rising above the diagnostic signal generated by the blood rouleaux.

Experimental results from the arterial simulator showed an increasing trend for increasing tensioning states of the arterial surrogate, as expected from the mathematical models in the scientific literature [22,23]. Moreover, PWV outcomes through ETiSIAM were compatible with the ones retrieved from LVDT signals for at least 67% of the stretching states applied (Table 10). In this case, the arterial surrogate is a compliant vessel in which the wall movement cannot be neglected, therefore constituting the main cause of the Doppler shift. This movement is related to the stiffness of the hose itself.

Hence, it is noteworthy that ETiSIAM could be adapted for the detection of physical phenomena of different nature, generating a Doppler shift. Therefore, the proposed method may also be a promising tool to overcome the current frame rate limits of US diagnostic systems in the detection of rapid time-varying phenomena in compliant vessels, such as pulse wave velocity. Moreover, the possibility to perform local measurements on the patient provides diagnostic information on the biomechanics of a local arterial wall [12,52], and such information can be used for early diagnosis of CVDs via the stiffness increment in the arterial wall. Conversely, regional assessments provide average and less accurate PWV measurements over a long segment, preventing the localization of arterial abnormalities since mechanical characteristics vary along the arterial segment [52].

Despite the moderate accuracy of the results that point to the need for further investigations, the proposed method offers an exploitable tool for non-invasive PWV estimation, making use of diagnostic instrumentation commonly used in clinical practice. Moreover, the dual application of ETiSIAM for the assessment of flow velocities and pulse wave velocities, through the pulse transit time estimation, is the main achievement of the present study, therefore resulting in a promising tool that could hopefully open up a new research field and applications in the industrial and biomedical fields. For example, the novel method hereby investigated could be useful for solving some problems encountered in the current scientific field regarding the non-invasive estimation of PWV [12], possibly stimulating the development of novel instrumentation for the diagnosis and monitoring of cardiovascular diseases. In a real-case scenario, heart rate variability may represent a real challenge for ETS reconstruction. Nevertheless, the preliminary study, carried out on real ECG recordings in which no arrhythmias were present, showed that the reconstruction method was robust enough despite the influence of the heart rate and amplitude variability [36]. It is worth noting that, despite the limitations found, the method can be improved on the basis of future experimental campaigns to be carried out on both healthy populations and pathological patients. Further investigations may also include cohorts with different environmental and lifestyle backgrounds, as in [15]. It will be expected that, in the clinical case, the two phenomena (i.e., arterial wall movement and echogenic blood flow) will both occur in a significant way that warrants further studies in the near future. The clinical outcomes will be crucial to defining and developing predictive, diagnostic and prognostic indices for CVDs.

Based on the promising results obtained, further studies are deemed necessary (a) to improve the image analysis method, including the investigation of different techniques for a more effective transit time assessment, (b) for an in-depth analysis of the uncertainty sources that could affect the estimation of both FAV and PWV, and (c) to test ETiSIAM on different ultrasound probe models. In particular, a calibration procedure may be applied to PWV outcomes retrieved through ETiSIAM to minimize any errors in clinical measurements, as shown in Figure 8. In addition, a further future development could include the design of a novel arterial simulator, based on a different technology for the non-contact transduction of the arterial surrogate radial displacement, in order to minimize the insertion errors in the PWV measurement. This limitation was mainly due to the mass of the cores and rods of the LVDT transducers. Finally, the possibility to test ETiSIAM on further arterial simulators, specifically those able to vary the stiffness of the surrogate with different physical principles from those used in the present study, is not excluded, as to allow a finer and more repeatable adjustment of the stiffness variation and, consequently, of the pulse wave velocity.

## 6. Conclusions

In recent years, statistical studies have highlighted the increasing incidence of cardiovascular diseases, drawing attention to the investigation of predictive parameters. Among them, pulse transit time is an accepted marker of cardiovascular health status and, although several studies have been carried out on this physical quantity, no image analysis methods have been developed. In this regard, the present study aims to provide a contribution by proposing a novel image analysis method based on equivalent time sampling for PTT estimation. To the authors’ knowledge, ETS has been applied in the research field as a sampling technique in the pre-acquisition phase and rarely used as a post-processing tool. Therefore, the proposed method post-processes color Doppler data acquired through an ultrasound diagnostic system from two different experimental setups, highlighting the dual application of ETiSIAM. From pulse transit time assessment, it allows the estimation of flow average velocities and pulse wave velocities, according to the mechanical characteristics of the vessel constituting the experimental setup.

## 7. Patents

Italian patent No. 102,021,000,005,042 filed on 4 March 2021 by University of Roma Tre. Title: “Sistema per la misura della velocità dell’onda sfigmica in vasi sanguini”.

## Figures and Tables

**Table 1:** Flow phantom technical characteristics.

Parameter	Characteristic
US phantom model	Doppler 403^TM^ flow phantom
TMM ^(1)^	patented high equivalence (HE) gel^TM^
TMM sound speed	1540 ± 10 m·s^−1^
BMF ^(2)^ sound speed	1550 ± 10 m·s^−1^
Pulse flow period	(0.5–4.0) ± 0.2 s
Flow range	(1.7–12.5) ± 0.4 mL·s^−1^
Tube inner diameter	5.0 ± 0.2 mm
Attenuation coefficient	0.70 ± 0.05 dB·cm^−1^·MHz^−1^
Dimensions	28.0 × 30.5 × 22.0 cm

^(1)^ TMM: tissue mimicking material; ^(2)^ BMF: blood mimicking fluid.

**Table 2:** Color Doppler configuration settings.

Parameter	Setting
Doppler frequency (MHz)	2.5
Field of view (cm)	FOV_ph_ = 10 ^(1)^ and FOV_as_ = 24 ^(2)^
Wall filter	minimum
Line density	maximum
Persistence	minimum
Edge enhancement	minimum
Transmitted power	maximum
Video duration (s)	140

^(1)^ FOV_ph_: field of view setting for the flow phantom experimental setup; ^(2)^ FOV_as_: field of view setting for the arterial simulator experimental setup.

**Table 3:** Arterial simulator technical characteristics.

Component	Characteristic	Value
Arterial surrogate	inner diameter	25 mm
	outer diameter	28 mm
	length	50 cm
Tensioning system	initial stretching displacement *y*_0_	7.0 cm
Analog manometer	full scale inner pressure	100 kPa
Water tank	dimensions	80 × 50 × 50 cm
	water height above the surrogate	17 cm
Low echogenic mixture	distilled water volume	5 L
	glycerin mass	1 kg
	alumina mass	50 g
NI-DAQ	sampling frequency	500 kHz
	number of channels	8
	ADC number of bits	16

**Table 4:** Arterial simulator settings.

Parameter	Setting
Stretching displacement range	0 to 1.5 cm
Number of tensioning states	6
Tensioning step	0.3 cm
Tests per tensioning state	6
Surrogate inner pressure	25 kPa
LVDT distance	45.5 cm
LVDT sampling frequency	2 kHz
Pump frequency	3.4 Hz
Water temperature	24 °C

**Table 5:** Method settings for PTT estimation through the two experimental setups.

Parameter	Flow Phantom	Arterial Simulator
Trigger signal	sinusoidal waveform	LVDT waveform
Trigger event	sinusoid peak	radial displacement peak
Pump frequency (Hz)	0.5, 0.8, 1.0, 1.3 and 2.0	3.4
Recording sites distance (cm)	*L_ph_* = 3	*L_as_* = 12

**Table 6:** MCS settings for the flow phantom experimental setup.

Parameter	Distribution	Mean Value ± SD
*L_ph_* ± *δ_px_* (mm)	Uniform	30.0 ± 0.1
*PTT_ph_* ± *σ*_Δ*t*,*ph*_ (ms)	Uniform	Δ*t_ph_* ± *σ*_Δ*t*,*ph*_

SD: standard deviation.

**Table 7:** MCS settings for the arterial simulator experimental setup.

Parameter	Distribution	Mean Value ± SD
*L_as_* ± *δ_px_* (mm)	Uniform	120.0 ± 0.3
*PTT_as_* ± *σ*_Δ*t*,*as*_ (ms)	Uniform	Δ*t_as_* ± *σ*_Δ*t*,*as*_

SD: standard deviation.

**Table 8:** Comparison between estimated and nominal FAV values (mean ± SD) according to the flow regime at 1.3 and 2.0 Hz pump frequencies.

Flow Rate (mL·s^−1^)	Pump Frequency (Hz)	Nominal FAV (cm·s^−1^)	Estimated FAV (cm·s^−1^)	*μ_SD_*_%_ (%)	*ε_FAV_*_%_ (%)
2.0	1.3	10.2 ± 2.8	10.4 ± 0.1	1.0	2.0
	2.0		10.1 ± 0.1	1.0	1.0
4.0	1.3	20.4 ± 2.8	20.0 ± 0.4	2.0	2.0
	2.0		19.3 ± 0.2	1.0	5.4
6.0	1.3	30.6 ± 2.8	30.3 ± 0.8	2.6	1.0
	2.0		29.1 ± 0.4	1.4	4.9
8.0	1.3	40.7 ± 2.8	37.9 ± 1.2	3.2	6.9
	2.0		37.2 ± 0.6	1.6	8.6
10.0	1.3	50.9 ± 2.8	47.2 ± 1.8	3.8	7.3
	2.0		48.2 ± 1.0	2.1	5.3

**Table 9:** Comparison between estimated and nominal FAV values (mean ± SD) according to the Doppler phantom pump frequencies for a pulsatile flow regime of 8.0 mL·s^−1^.

Pump Frequency (Hz)	ETS Frequency (Hz)	Nominal FAV (cm·s^−1^)	Estimated FAV (cm·s^−1^)	*μ_SD_*_%_ (%)	*ε_FAV_*_%_ (%)
0.5	35.5	40.7 ± 2.8	38.8 ± 10.6	27.3	4.7
0.8	88.8		38.0 ± 3.4	8.9	6.6
1.0	143.0		37.9 ± 2.1	5.5	6.9
1.3	267.8		37.9 ± 1.2	3.2	6.9
2.0	594.0		37.2 ± 0.6	1.6	8.6

**Table 10:** Comparison between PWV outcomes (mean ± SD) retrieved through the arterial simulator experimental setup for each tensioning state.

Tensioning State (cm)	*PWV_LVDT_* (m·s^−1^)	*PWV_ETS_* (m·s^−1^)	*μ_PWV_*_,*SD*%_ (%)	*ε_PWV_*_%_ (%)
*y*_0_	8.4 ± 0.2	7.3 ± 0.5	6.8	13.1
*y*_0_ + 0.3	8.7 ± 0.3	7.9 ± 0.8	10.1	9.2
*y*_0_ + 0.6	9.3 ± 0.3	8.9 ± 0.8	9.0	4.3
*y*_0_ + 0.9	9.6 ± 0.4	9.5 ± 0.9	9.5	1.0
*y*_0_ + 1.2	10.7 ± 0.3	11.1 ± 0.6	5.4	3.7
*y*_0_ + 1.5	11.1 ± 0.3	13.9 ± 1.4	10.1	25.2

*y*_0_: initial stretching of the arterial surrogate; *PWV_LVDT_*: pulse wave velocity values retrieved with the displacement transducers; *PWV_ETS_*: pulse wave velocity values retrieved with ETiSIAM.

## Data Availability

Not applicable.
